# The Treatment of Fractures

**Published:** 1901-10

**Authors:** 


### BOOK REVIEWS.

THE TREATMENT OF FRACTURES. By W. L. ESTES, A. M., M. D., Director and Physician and Surgeon-in-Chief of St. Luke's Hospital, South Bethlehem, Pa. With numerous original illustrations. New York: International Journal of Surgery Co. 1900. [Price, $2.00.]

The International Journal of Surgery in publishing this book has given to the profession a handy and thoroughly practical volume on fractures.

Dr. Estes shows by his treatment of the subject that he is master of it and has made a most valuable contribution to surgical literature. True, it is not pretentious, nor is it bulky—there are only 216 pages all told—but it is full of the meat of the subject and the illustrations tell stories of their own.

Dr. Estes calls his work a "series of papers." Whatever they are, whether a series of papers, a string of lectures, or what not, they are veritable gems. He speaks from a wide and successful practice and one feels intuitively as the pages are gone over that there is something original, and not a rehash of an already rehashed book. Not that Dr. Estes is narrow-minded and egotistical to the extent of worshipping exclusively at his own shrine; far from it. He has quoted liberally and freely from modern authorities and he has taken from them their best ideas and suggestions. Yet the bulk of the work is original and the result of fifteen years' hospital experience in a railroad and mining district.

In the main the discussion of the various fractures is confined to the treatment, and only when it is necessary to a clear understanding of the treatment to be followed are the mechanics touched upon. This is one of the many features which make the work one of practical value. Monographic in tone and treatment, Estes' fractures is worth looking over, even if one thinks he knows all about fractures. N. W. W.

